# Protein lipoylation in cancer: metabolic reprogramming and therapeutic potential

**DOI:** 10.1038/s41420-025-02718-z

**Published:** 2025-09-02

**Authors:** Sainan Li, Yingchao Liu, Wanye Hu, Aoli Deng, Xueying Ren, Lulu Chen, Yajuan Lu, Yunyi Wu, Hangqi Huang, Jinghao Cao, Jing Du, Jun Xia, Yanchun Li

**Affiliations:** 1 Laboratory Medicine Center, Department of Clinical Laboratory, Zhejiang Provincial People’s Hospital (Affiliated People’s Hospital), Hangzhou Medical College, Hangzhou, Zhejiang China; 2 Department of Clinical Laboratory, The Second Affiliated Hospital of Soochow University, Suzhou, Jiangsu China; 3 Department of Clinical Laboratory, The Second Affiliated Hospital of Zhejiang Chinese Medical University, Hangzhou, Zhejiang China; 4 Department of Clinical Laboratory, Affiliated Hangzhou First People’s Hospital, School of Medicine, Westlake University, Hangzhou, Zhejiang China

**Keywords:** Cancer metabolism, Post-translational modifications

## Abstract

Protein lipoylation, a mitochondria-specific post-translational modification (PTM) evolutionarily conserved from bacteria to mammals, plays critical role in metabolic processes. In humans, four identified lipoylated proteins serve as essential components of key enzymes involved in glycolysis, the tricarboxylic acid (TCA) cycle, and amino acid metabolism. The dynamic addition or removal of lipoylation modifications critically regulates the functional activity of these enzymes, with dysregulation strongly associated with cancers. Notably, cancer-associated metabolic reprogramming frequently coincides with functional impairment of lipoylated proteins, which subsequently modulates tumor growth through metabolic adaptation. In this review, we systematically summarized the biosynthesis of lipoic acid (LA), introduced the basic structure of lipoylated protein and presented the regulation of lipoylation. Since metabolic reprogramming is an important feature of tumorigenesis, we discussed the relationship between protein lipoylation and tumor metabolic reprogramming. Cuproptosis is a novel form of cell death characterized by copper-mediated lipoylation, which disrupts mitochondrial metabolism and induces cell death through the aggregation of lipoylated proteins in the TCA cycle. We highlighted the therapeutic potential of targeting lipoylation to disrupt cancer cell energy metabolism, particularly through cuproptosis. These insights reveal the intricate interplay between lipoylation and cancer progression and open new avenues for developing targeted therapies. Furthermore, we proposed innovative combinatorial strategies leveraging the crosstalk between cuproptosis and ferroptosis to overcome tumor drug resistance. These insights establish lipoylation as a promising therapeutic axis for developing precision cancer therapies targeting metabolic vulnerabilities.

## Facts

- This article systematically reviews the structures and functions of the four human lipoylated proteins (PDH, KGDH, BCKDH, and GCS), which collectively regulate metabolic nodes to influence cancer cell energetics and biosynthesis.
- The study elucidates how protein lipoylation plays a pivotal role in cancer metabolic reprogramming, with its dynamic regulation being linked to glycolysis, TCA cycle function, and amino acid metabolism in malignant cells.
- FDX1 modulates cancer cell susceptibility to cuproptosis by controlling lipoylation levels. Targeting the activity level or modification of these enzymes can precisely intervene in the metabolic weakness of cancer cells, providing a new strategy for overcoming drug resistance and developing combination therapies.

## Questions

- SIRT2 and SIRT4 are recognized as delipoylases, but their cancer-specific regulatory roles and effects on other lipoylated proteins (KGDH, BCKDH, GCS) remain unclear.
- Cancer cells (e.g., TNBC, AML, HCC) exhibit metabolic plasticity by switching between glycolysis and OXPHOS. Given that lipoylated proteins play a crucial regulatory role in this metabolic flexibility, could targeted lipoylation represent a more effective strategy for disrupting cancer metabolism?
- The central role of GSH as a metabolic nexus between ferroptosis and cuproptosis raises a question of whether these two distinct cell death pathways synergistically induce tumor cell death.

## Introduction

Concerns about cancer remain unabated as one of the world’s major public health problems. According to global statistics for 2022, there will be nearly 20 million new cases of cancer and 10 million cancer-related deaths worldwide. Demographic-based projections suggest that by 2050, the number of new cancer cases will reach 35 million [1]. Despite advances in diagnosis and treatment techniques, the overall survival rate of cancer is still limited, and there is an urgent need to continue to explore new treatment strategies. Cancer metabolic reprogramming, as a core hallmark of cancer, has received extensive attention. Cancer metabolic reprogramming involves enhanced glycolysis, aberrant de novo lipid synthesis, and overactivation of key metabolic pathways such as glycine, glutamine, and branched-chain amino acids, which work together to drive tumor proliferation [2–4]. Since Otto Warburg’s pioneering observations on aerobic glycolysis, targeting dysregulated metabolism has emerged as a promising therapeutic strategy. Traditional targets such as glucose transporters, glycolytic enzymes, glutaminase inhibitors, and oxidative phosphorylation (OXPHOS) inhibitors, despite showing clinical potential, often face limitations due to compensatory metabolic adaptations and off-target toxicities [4, 5]. The discovery of cuproptosis in 2022 unveiled a novel vulnerability in cancers with high OXPHOS activity [6]. Central to this process is lipoylation, a conserved post-translational modification (PTM) critical for mitochondrial enzyme function. Lipoylated proteins serve as direct targets of cuproptosis, and enhancing lipoylation sensitizes cells to copper-induced death [6, 7]. This review elucidates how leveraging the lipoylation-cuproptosis axis represents a paradigm shift in metabolic targeting, addressing the limitations of conventional approaches through its unique mechanism and tumor selectivity.

Protein lipoylation is a vital PTM that involves the covalent attachment of lipoic acid (LA) to the lysine residues of specific proteins [8]. This modification occurs primarily in enzymes involved in mitochondrial energy metabolism, such as the pyruvate dehydrogenase complex (PDH) and the α-ketoglutarate dehydrogenase complex (KGDH) [9]. Protein lipoylation is highly evolutionarily conserved, and the proteins involved in or modified by lipoylation are predominantly found in the core metabolic pathways of bacteria and mammals [10]. Only four lipoylated proteins have been identified in mammals, involving PDH, KGDH, branched-chain α-ketoacid dehydrogenase complex (BCKDH), and glycine cleavage system (GCS) [11]. PDH, KGDH, and BCKDH belong to the α-ketoacid dehydrogenase complex family, which catalyzes the oxidative decarboxylation of specific α-ketoacids while producing the corresponding acyl-Coenzyme A (acyl-CoA) product and reducing nicotinamide adenine dinucleotide (NAD^+^) to reduced form of nicotinamide adenine dinucleotide (NADH) [12]. PDH and KGDH are a part of the TCA cycle, and lipoylation is critical for the activity of these two complexes [10]. BCKDH is a rate-limiting enzyme in branched-chain amino acid (BCAA) catabolism [13], and GCS is involved in the biosynthesis of several key intermediates in one-carbon metabolism [14]. Acetoin dehydrogenase (AODH), the fifth lipoylation complex found in bacteria, breaks acetylaminoyl energy storage molecules into acetyl-CoA and acetaldehyde [15]. Due to the central role of lipoylated protein in the TCA cycle and amino acid metabolism, its dysfunction or expression changes profoundly affect the metabolism of cancer cells. Therefore, targeting these lipoylated proteins and their related pathways has become an important strategy for regulating the growth and survival of cancer cells. The purpose of this article is to systematically review the latest research progress on the alteration of lipoylated protein in cancer and its regulatory mechanism, and to look forward to future research directions in this field.

## Research Progress of Protein Lipoylation

### Biochemical mechanisms of protein lipoylation

The study of lipoylation modification as a key bridge between metabolism and protein function can be traced back to the 50 s of the 20th century. For the first time, the Reed lab isolated lipoic acid from the PDH complex and demonstrated the necessity of lipoylation modification for enzymatic reactions [16]. Subsequently, Cronnan’s team elucidated the de novo synthesis pathway of lipoic acid in *Escherichia coli* and the role of protein ligase [17, 18]. These pioneering works provide a theoretical foundation for the subsequent research of lipoylation in metabolic diseases, cancer, and other fields.

Lipoylation involves the covalent attachment of LA to specific proteins [19]. In mammals, there are two forms of LA: exogenous LA and endogenous LA. Endogenous LA is covalently bound to lipoylated proteins, which is obtained through dietary supplements, and is mainly present in the blood and cytoplasm in free form to exert antioxidant functions [20]. Endogenous LA is synthesized in mitochondria by inserting sulfur atoms at carbon 6 and 8 with intracellular octanoic acid (OA) as substrate [21]. OA is the primary product of the mitochondrial type II fatty acid synthesis pathway (mtFASII) [22]. Endogenous LA does not exist in a free form, but is directly transferred to the lipoylated protein as an intermediate in an enzymatic reaction [23–25]. The lipoylation pathway of protein in *Escherichia coli* is the most widely studied. *Escherichia coli* has two protein lipoylation pathways: the de novo synthesis and salvage synthesis pathways, which complement each other [26–28]. The de novo synthesis pathway uses acyl carrier protein (ACP), an intermediate product of fatty acid synthesis and metabolism, as a substrate [26]. The caprylyl group is first transferred to the target protein by octanoyl transferase LipB, followed by insertion of two sulfur atoms into the octanoyl group by lipoyl synthase (LipA), completing the lipoylation modification [29]. The salvage pathway utilizes lipoate-protein ligase (LplA) to mediate a two-step lipoylation process: first, LplA catalyzes the ATP-dependent activation of exogenous LA to generate lipoyl-AMP; subsequently, it facilitates the transfer of the lipoyl moiety from this activated intermediate to specific target proteins, thereby completing the lipoylation modification [30]. It is important to note that the de novo synthetic pathway is prevalent in bacteria, whereas the salvage pathway is only found in specific bacterial species like *Escherichia coli, Bacillus subtilis, Staphylococcus aureus*, etc [11, 28]. Mammalian cells are deficient in LplA and cannot use the salvage pathway [27, 31].

**Fig. 1:**
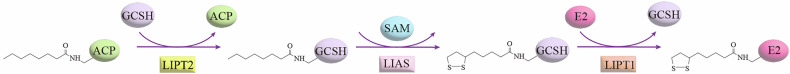
Human protein lipoylation pathway. In the process of human protein lipoylation, LIPT2 transfers the octanoyl group of octanoyl-ACP to GCSH. Subsequently, LIAS catalyzes the radical-mediated insertion of two sulfur atoms into the C-6 and C-8 positions of the octanoyl group. Finally, LIPT1 transfers the lipoyl group from the GCSH protein to the E2 subunit of the lipoylated protein, thereby completing lipoylation modification.

Human mitochondria contain their own de novo lipoic acid synthesis pathway, involving enzymes homologous to LipB and LipA, namely lipoic acid transferase 2 (LIPT2), lipoic acid synthase (LIAS), and lipoic acid transferase 1 (LIPT 1) [24, 31]. The process occurs via the following steps (Fig. 1). First, FAS II synthesizes octanoyl-ACP using ACP and OA as substrates [30]. Catalyzed by LIPT2, the octanoyl group is transferred to the carrier protein H of the glycine cleavage system (GCSH) [32]. In the second step of lipoylation, LIAS utilizes iron-sulfur clusters and S-adenosylmethionine (SAM) to generate the 5’-deoxyadenosyl radical (5’-dA). The 5’-dA radical sequentially removes one hydrogen atom from each of the C6 and C8 positions of the OA, allowing for subsequent sulfur insertion [30, 31, 33]. Finally, LIPT1 completes the lipoylation reaction by transferring LA from the H protein to a modified protein [34]. In these multienzyme complexes, specific components (GCSH) are modified by covalently attaching LA to the ε-amino group of the conserved lysine residues via amide bonds [35]. The modified lysine residue protrudes from the surface of a highly conserved domain, often referred to as the lipoyl domain (LD) [36]. LD acts as a “swinging arm,” shuttling reaction intermediates between multiple active sites of these multiprotein complexes as a covalent substrate channel [37].

### Lipoylated protein structure and mitochondrial metabolism

**Fig. 2:**
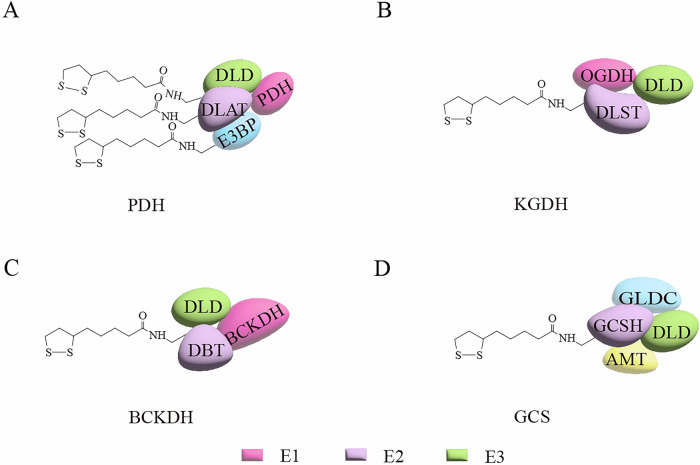
Lipoylated protein complex in humans. In humans, four mitochondrial enzyme complexes are known to be lipoylated: PDH (**A**) KGDH (**B**) BCKDH (**C**) and GCS (**D**). Among them, the DLAT subunit of PDH contains two LDs, and E3BP contains one. KGDH, BCKDH, and GCS each contain one LD, which are located on the DLST, DBT, and GCSH subunits.

Currently, the three lipoylated proteins discovered in humans belong to the α-ketoacid dehydrogenase complex family, including PDH, KGDH, and BCKDH, which are involved in mitochondrial metabolism [38]. All α-ketoacid dehydrogenase complexes have a substantially similar structure and are composed of multiple copies of three distinct subunits (E1–E3) [39] (Fig. 2). The E2 subunit forms the core of the complex structure, and the flexible lipoylation arm of E2 interconnects the active sites of the E1 and E3 subunits [36]. The E3 subunit in all α-ketoacid dehydrogenase complexes is identical [40]. The carboxyl group of lipoic acid is covalently bound to the ε-amino group on the lysine residue in the conserved domain of the E2 subunit, forming the lipoyl domain. Another lipoylated GCS protein contains four subunits: the P protein (glycine decarboxylase, GLDC), H protein (glycine cleavage system H protein, GCSH), T protein (aminomethyltransferase, AMT), and L protein (dihydrolipoamide dehydrogenase, DLD) [41] (Fig. 2). GCSH has a lipoylation site central in the glycine cleavage reaction because its prosthetic group LA continuously interacts with the other three components of the complex [42].

#### PDH

Human PDH is a multienzyme complex with three subunits: E1, E2, E3, and E3-binding protein (E3BP) [43] (Fig. 2A). The core of the mammalian PDH complex is a 60-subunit icosahedral structure assembled from E2 and E3BP subunits, with the E3 protein dynamically anchored to the surface of this core [44]. E3BP is homologous to E2 but has no catalytic function [45]. Pyruvate dehydrogenase (PDH, E1 subunit) is a thiamine pyrophosphate-dependent decarboxylase that catalyzes the irreversible decarboxylation of pyruvate to form the hydroxyethyl-TPP intermediate [46]. The E2 subunit (dihydrolipoyllysine-residue acetyltransferase, DLAT) contains two LDs, whose function highly depends on the covalent modification of LA [47]. E2 transfers the acetyl group from LA to coenzyme A (CoA), forming acetyl-CoA, while LA is concurrently reduced to dihydrolipoamide [48]. Dihydrolipoamide transfers two electrons to the flavin adenine dinucleotide (FAD) in E3 (DLD), generating FADH₂ [49]. Afterwards, FADH₂ donates these electrons to NAD⁺, producing NADH, while dihydrolipoamide is regenerated to its oxidized form, thereby completing the catalytic cycle [50]. PDH activity is affected by multiple regulatory mechanisms, the most extensively studied of which are phosphorylation and dephosphorylation of the EI subunit. Four isoforms of pyruvate dehydrogenase kinase (PDK1-4) can phosphorylate the E1 subunit of PDH, resulting in PDH inactivation and inhibition of the mitochondrial TCA cycle [51]. Two isoforms of pyruvate dehydrogenase phosphatase (PDP1 and PDP2) dephosphorylate the E1 subunit of PDH and restore the catalytic activity [52].

A core feature of metabolic reprogramming in cancer is an increased dependence on mitochondrial metabolism to meet its dual energy and biosynthetic needs. In this process, the DLAT-mediated conversion of pyruvate to acetyl-CoA is a critical rate-limiting step in bridging glycolysis with the TCA cycle [53]. Acetyl-CoA is not only a TCA cycle fuel, but also a key precursor for de novo synthesis of fatty acids [54]. The rapid proliferation of cancer cells is extremely dependent on the activation of the de novo fatty acid synthesis pathway [55]. Therefore, DLAT plays an indispensable role in supporting the abnormally strong anabolic demand and energy supply of cancer cells.

#### KGDH

KGDH is composed of three protein subunits: 2-oxoglutarate dehydrogenase (OGDH, E1 subunit), dihydrolipoyllysine residue succinyltransferase (DLST, E2 subunit), and DLD (E3 subunit) [56] (Fig. 2B). The DLST component is a multi-domain polypeptide chain that aggregates into either icosahedral (60-subunit) forms in eukaryotes or octahedral/cuboctahedral (24-subunit) forms in bacteria, depending on the source [36]. DLST is the core subunit of KGDH, which contains a LD in its structure [57]. This domain is connected to the enzyme’s catalytic core domain via a flexible peptide segment [58]. These three subunits of the KGDH complex work synergistically, starting with E1, which catalyzes the oxidative decarboxylation of α-KG to form CO_2_ and a covalently bound succinimide group [59]. DLST transfers the succinyl group produced by E1 to coenzyme A to produce succinyl-CoA [60]. The DLST subunit contains an LD that receives a succinyl group from the E1 subunit and transfers it to CoA to generate succinyl-CoA [61], completing the second step of the KGDH catalytic process [62]. The E3 subunit oxidizes the reduced LA cofactor in the E2 subunit and transfers electrons to NAD^+^ to generate NADH [61].

DLST, as the core subunit of KGDH, catalyzes the oxidation of α-KG to succinyl-CoA, a key intermediate in the TCA cycle [63]. This is a critical step in the TCA cycle and directly impacts the efficiency of ATP generation. In addition, DLST acts as a bridge between nitrogen and carbon metabolism. Glutamine metabolic reprogramming is a typical feature of tumor metabolism [64, 65]. As an essential source of nitrogen and energy for tumor cells, glutamine uptake and metabolism are significantly increased in cancer cells to support protein and nucleic acid synthesis, promoting the continued growth and proliferation of tumor cells [66, 67]. KGDH catalyzes the conversion of α-KG produced by glutamine breakdown to succinyl-CoA, allowing it to enter the TCA cycle [68]. DLST connects the glutamine and TCA cycles through an irreplaceable catalytic reaction and is the central hub for integrating glutamine metabolism into the TCA cycle. This process drives the TCA cycle to maintain the supply of ATP, which is the backbone of energy metabolism in cancer.

Studies have demonstrated significant differences in the dependence of different cell subsets on TCA cycle in triple-negative breast cancer (TNBC). DLST-dependent TNBC cell subsets are highly expressed with DLST, and glutamine is preferentially used to promote tumor cell growth and invasion [69]. It provides a new idea for the treatment of DLST-dependent TNBC subsets, that is, to reduce the tumor burden and aggressiveness of TNBC by inhibiting DLST or its related metabolic pathways. The expression of DLST was significantly upregulated in MYC-over-driven T-cell acute lymphoblastic leukemia (T-ALL) and primary T-ALL patient samples. DLST knockdown disrupts the TCA cycle by disrupting the breakdown of α-KG, ultimately triggering cancer cell death [70]. Targeting specific steps in the DLST or TCA cycle is expected to be a promising metabolic therapy strategy for MYC-mediated leukemia.

#### BCKDH

BCKDH is a multienzyme complex responsible for catabolizing branched-chain amino acids (leucine, isoleucine, and valine) [71]. The structure of BCKDH is similar to that of PDH and usually consists of three subunits [72] (Fig. 2C). The E1 subunit is branched-chain α-ketoacid dehydrogenase (BCKDH) containing BCKDHA and BCKDHB heterotetramers, the E2 subunit is dihydrolipoyl transacylase (DBT), and the E3 subunit is DLD [73, 74]. The human BCKDH complex is organized around a cuboctahedral 24-mer core of E2 subunits, which provides the structural and catalytic scaffold for docking E1 and E3 components [75]. The E2 subunit usually contains an LD that can freely oscillate between E1, E2, and E3, passing reaction intermediates from one active center to another and facilitating the enzymatic reaction [12].

BCAAs are essential nutrients for tumor growth, involved in multiple biosynthetic pathways, and act as a source of energy [76]. In the first step of BCAA catabolism, the amino groups of BCAA are first transferred by branched-chain amino acid transaminase (BCAT) to α-KG to produce the corresponding branched-chain ketoacids (BCKA) and glutamic acid [77]. The BCKDH complex in the mitochondrial matrix for the irreversible oxidative decarboxylation of BCKA and the production of branched-chain acyl-CoA intermediates catalyzes the second step in BCAA catabolism [78]. Finally, E3 transfers the released electrons to NAD⁺ for the production of NADH [79]. BCAA catabolism is completed, and BCAA eventually enters the TCA cycle or other metabolic pathways.

The BCKDH complex is a rate-limiting enzyme in the metabolism of BCAA, which means that regulating its activity is of great significance for the homeostasis of BCAA and branched α-ketoacids in vivo. BCKDH activity is tightly regulated by branched chain keto acid dehydrogenase kinase (BCKDK) and protein phosphatase magnesium-dependent 1 A, kinase (PPM1K) [80]. BCKDK phosphorylates the E1 subunit of BCKDH at Ser 293 (also known as site 1) and Ser303 (site 2) and inhibits the activity of BCKDH [72]. In contrast, PPM1K dephosphorylates BCKDK at the same site to restore the BCKDH activity [79]. Current understanding of how lipoylation modulates the activity of the branched-chain α-ketoacid dehydrogenase (BCKDH) complex remains limited, with few studies systematically investigating this regulatory mechanism.

The metabolism of BCAA is significantly enhanced in various cancers, including lung cancer, colorectal cancer (CRC), pancreatic cancer, and leukemia, among others [81–84]. This abnormality leads to the accumulation of BCAA, especially leucine, in cells, which continuously activates the mammalian target of rapamycin complex 1 (mTORC1) signaling pathway, thereby driving tumor growth and proliferation [85]. The inhibition of BCKDH activity is a key regulatory node in this process. Elevated expression of BCKDK has been observed in human hepatocellular carcinoma (HCC), resulting in the phosphorylation and subsequent inactivation of BCKDH complex [86]. Although current research on DBT in the field of cancer is still insufficient, given its critical role in the catalytic process of the BCKDH complex, targeting DBT is expected to open up new strategies for cancer treatment. An in-depth understanding of BCAA metabolic abnormalities and their regulatory mechanisms reveals fundamental mysteries of tumor metabolism. Furthermore, it provides crucial theoretical foundations and identifies clear target directions for developing novel cancer diagnostic markers and therapeutic strategies.

#### GCS

Another lipoylated protein, GCS, is a multienzyme complex that breaks down glycine and provides one carbon unit for tetrahydrofolate (THF) [87, 88]. GCS is loosely bound to the inner mitochondrial membrane in plants and animals [89]. Vertebrate H protein comprises 125 amino acid residues, and LA is covalently linked to Lys-59 residue to form the lipoyl lysine arm [90]. The lipoyl lysine arm of GCSH is often folded in β barrels, a common feature observed in similar domains in different species [91]. GCSH, as the carrier protein of lipoic acid, does not play an enzymatic role in the cleavage of glycine but shuttles between the active sites of the other three components of GCS as a reaction intermediate and a reduction equivalent [92]. Specifically, the P protein catalyzes the decarboxylation of glycine and the sulfhydryl group formed by disulfide bond cleavage in the lipoate attached to GCSH to form an aminomethyl lipoate intermediate [92](Fig. 2D). In THF-rich cells, the T protein further catalyzes this intermediate, producing ammonia, N^5^, N^10^-methylene-H4folate, and H-protein with reduced lipoate [93]. Finally, the L protein oxidizes the reduced lipoate attached to GCSH and generates a reduced equivalent NADH [42, 94]. It is worth mentioning that this function of GCSH relies on the covalent modification of the cofactor LA to form the active form of GCSH [95]. Studies have shown that the lipoylated H protein (Hlip) is the active form, and the unlipoylated apo-form of the H protein (Hapo) inhibits the overall activity of GCS [42]. Existing studies have shown that increased expression of GCSH in *Arabidopsis thaliana* enhances the activity of GCS [96]. In addition, loss of GCSH function led to the death of mouse embryos before the second trimester of pregnancy [95]. Elevated plasma glycine in patients with nonketotic hyperglycinemia was associated with decreased GCSH activity and deficiency in the lipoic acid moiety [97].

The above research indicates that lipoylation affects the activity of H protein, thereby influencing the overall function of GCS. It was found that there were significant differences in the expression of serine/glycine metabolism-related proteins in thyroid cancer cell subtypes. Specifically, these proteins are expressed at higher levels in papillary thyroid carcinomas (PTCs) and poorly differentiated carcinomas (PDCs), while lower levels are expressed in medullary carcinoma (MC) [98]. These differences may be related to the aggressiveness and metabolic activity of tumors, and may also provide new ideas for targeted therapy [98]. In addition, GCSH is also highly expressed in breast cancer and non-small cell lung cancer (NSCLC) [99]. GCSH and its lipoylation modification are expected to be an important regulatory node and potential therapeutic target in cancer metabolic reprogramming. In conclusion, these four lipoylated enzyme complexes profoundly shape the metabolic network of cancer cells by coordinately regulating the activity of key metabolic nodes. They serve as a pivotal basis for understanding cancer metabolic mechanisms and developing novel therapeutic strategies. The table below provides a summary of the subcellular localization of four human lipoylated proteins and their main metabolic functions (Table 1).

**Table 1:** Localization of four human lipoylated proteins and their main functions.

Lipoylation protein	Modified subunits	Intracellular localization	Main functions:
PDH	DLAT E3BP	Inner mitochondrial nucleus [12] Inner mitochondrial [193]	Catalyzes the conversion of pyruvate to acetyl-CoA [53]. Anchor other subunits of the PDH complex [45].
KGDH	DLST	Inner mitochondrial nucleus [12]	Catalyzes the conversion of α-ketoglutarate to succinyl-CoA [194].
BCKDH	DBT	Inner mitochondrial [195]	Catalyzes the catabolism of branched-chain amino acids [196].
GCS	GCSH	Inner mitochondrial membrane [92]	Oxidizes glycine and provides a one-carbon unit for tetrahydrofolate [41].

## The Dynamic Regulation of Lipoylation

### Negative regulation of lipoylation - Delipoylation

**Fig. 3:**
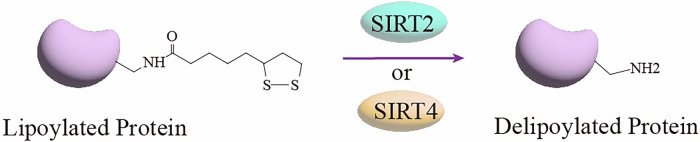
Delipoylation by SIRT2 or SIRT4 in humans. The process in which SIRT2 or SIRT4 catalyzes the lipoylated protein to lose the lipoyl group and be converted into a delipoylated protein.

Although protein lipoylation is important in regulating mitochondrial metabolism, there are few studies on its regulatory mechanisms, especially in eliminating lipoylation modifications. Delipoylation refers to the process of removing LA from protein lysine residues. The first enzyme with this function, lipoamidase, was identified in *Enterococcus faecalis* [100]. Mitochondrial sirtuin 4 (SIRT4) was recently discovered as a mammalian lipoamidase [101]. Human sirtuins (SIRT1-7) are a class of NAD^+^-dependent protein deacetylases that remove various acyl-lysine modifications [102]. Mitochondrial SIRT4 has little deacetylase activity but high activity against lipoylated and biotinylated substrates [103]. Mathias et al. determined using in vitro, cellular, and animal mouse models that SIRT4 could inhibit PDH activity by hydrolyzing the lipoamide cofactor in the DLAT subunit [104]. The activity and substrates of SIRT4 appear to be evolutionarily conserved, as the sirtuin homolog CobB in *Escherichia coli* and the sirtuin homolog SrtN in Gram-positive *Bacillus subtilis* can also act as lipoamidases that can deliver and inhibit the activity of PDH and KGDH [105]. In addition, SIRT4 has been shown to interact with other lipoylated multienzyme complexes, such as KGDH, BCKDH, and GCS. However, there is a lack of experimental studies on the delivery of these enzymes by SIRT4 [106]. Previous studies have demonstrated that SIRT2 is primarily localized in the cytoplasm and nucleus and has been shown to target histone and non-histone acetylated protein substrates for deacetylation [107]. Recent studies have shown that SIRT2 can also localize to mitochondria and interact directly with mitochondrial proteins [108]. To investigate the potential activity of SIRT2 on mitochondrial proteins, Xie et al. used chemical probes to show that SIRT2 can effectively catalyze the delipoylation of DLAT in cells and downregulate PDH activity [109]. Moreover, the lipoamidase activity of SIRT2 in vitro is much better than that of SIRT4, and SIRT2 is regarded as a powerful biomarker of protein lipoylation [109]. The following figure depicts the delipoylation process by human SIRT2 and SIRT4 (Fig. 3). Recent studies have made significant strides in identifying and preliminarily characterizing the functional roles of delipoylation. However, there are still many shortcomings, which provide rich directions for future research, including in-depth exploration of the mechanism of delipoylation and a comprehensive understanding of the role of lipoylation regulatory mechanisms in cellular metabolism.

### Genetic defects and metabolic abnormalities of lipoylation regulatory enzymes (LIPT1/2, LIAS)

The enzymes encoded by the LIPT1, LIPT2, and LIAS genes are involved in lipoylation. Defects in these enzymes reduce the activity of lipoylated mitochondrial enzymes, leading to metabolic disorders. Patients with LIPT2 mutations have been found to present with severe neonatal encephalopathy, decreased PDH and α-KGDH activity, abnormal leucine metabolism, and reduced protein lipoylation in LIPT2-mutated fibroblasts [32]. Similarly, LIAS deficiency, a key enzyme in lipoylation, significantly reduces the lipoylation of mitochondrial proteins, and patients with LIAS enzyme deficiency typically present with impaired energy metabolism, lactic acidosis, and hyperglycemia [110, 111]. Mutations in the LIPT1 gene affect the attachment of LA to PDH and KGDH, leading to reduced activity of these two key enzyme complexes [112]. Their decreased activity directly impacts the function of the mitochondrial respiratory chain, resulting in energy metabolism disorders. Notably, while LIPT1 is theoretically implicated in BCKDH lipoylation, the functional consequences of LIPT1 deficiency on BCKDH modification remain uncharacterized [112]. This suggests that further research is needed to investigate the role of LIPT1 in the lipoic acylation of BCKDH to better understand its pathophysiological significance in metabolic diseases.

### Other lipoylation regulatory factors

FDX1 is an iron-sulfur cluster protein involved in intracellular electron transfer that regulates a variety of metabolic processes [113]. Recent studies have shown that FDX1 is an upstream regulator of lipoylation, and FDX1 directly binds to LIAS to provide electrons to initiate a free radical chain reaction catalyzed by LIAS to regulate the lipoylation of intracellular proteins [114, 115]. GCSH serves as the core hub for octanoyl group transfer in mammalian mitochondria. Studies have shown that GLDC knockout blocks the GCSH-mediated transfer of dihydrolipoamide to the E2 subunits of PDH and KGDH, thereby leading to reduced lipoylation and activity of PDH and KGDH [116]. Methionine, a sulfur-containing amino acid, serves as the precursor of SAM [117]. Studies have shown that in yeast mitochondria, methionine restriction leads to a decrease in SAM levels, which in turn limits the lipoylation of mitochondrial enzymes [118]. This ultimately causes acetyl-CoA and α-KG to be diverted from the TCA cycle for the synthesis of amino acids such as arginine [118]. Polymerase-δ interacting protein 2 (POLDIP2) is a multifunctional nuclear-encoded mitochondrial protein, whose expression is downregulated under hypoxic conditions [119, 120]. Downregulation of POLDIP2 expression induces Clp-protease complex-mediated degradation of Ac-CoA synthetase in the middle-chain family member 1 (ACSM1), thereby inhibiting lipoylation of PDH and KGDH complexes [121]. In the high-glycolytic phenotype cell line BT549 of TNBC, overexpression of POLDIP2 was observed to significantly increase protein lipoylation and mitochondrial respiration, while accompanied by a marked inhibition of cell growth [121].

The dynamic regulation of lipoylation, through the collaborative actions of delipoylation enzymes, lipoylation-related synthetic enzymes, and metabolic factors, finely modulates the activity of mitochondrial enzymes and the flux of the TCA cycle, thereby influencing energy metabolism and cell fate. Lipoylation imbalance caused by genetic defects or environmental stimuli can trigger metabolic disorders and participate in tumor metabolic reprogramming. It is suggested that targeting the lipoylation regulatory network holds potential as a therapeutic strategy for metabolic disorders and cancer.

## Metabolic Reprogramming in Cancer Cells via Lipoylated Proteins

Cancer cells reprogram their metabolism to meet growth and proliferation demands. This process, known as cancer metabolic reprogramming, is a hallmark of cancer [122, 123]. Metabolic reprogramming involves many processes, including glucose, lipid, and amino acid metabolism [124]. The most widely studied form of glucose metabolic reprogramming is the Warburg effect. The Warburg effect, also known as aerobic glycolysis, refers to the process by which cancer cells rely on glycolysis instead of OXPHOS to produce energy and metabolic intermediates under aerobic conditions [125]. Nearly a century after the first observation of changes in cancer cell metabolic characteristics, key nodes targeting cancer cell metabolism have shown strong potential as an anticancer research direction.

The Warburg effect is a distinctive feature of metabolic reprogramming in cancer. Although this mode of metabolism is less efficient in producing ATP, it can quickly produce energy to meet the needs of rapid proliferation of cancer cells [126]. At the same time, glycolysis can also produce a large number of intermediate metabolites, such as ribose-5-phosphate, a product of the pentose phosphate pathway, for nucleotide synthesis [127]. While the Warburg effect represents a hallmark of cancer metabolism, certain malignancies—particularly those with dysregulated oncogene or tumor suppressor gene expression—maintain functional TCA cycle activity for energy production [128]. Emerging evidence reveals that specific cancer types, including glioblastoma, leukemia stem cells, and non-small cell lung cancer, can preferentially utilize OXPHOS under particular microenvironmental or genetic contexts [129–131]. However, therapeutic targeting of mitochondrial electron transport chain complexes remains pharmacologically challenging due to their essential roles in normal physiology.

In addition to glucose, cancer cells meet their biosynthetic and energy requirements by abnormally enhancing the uptake and metabolic reprogramming of a variety of amino acids [132]. Among them, glycine, glutamine and BCAAs are the core metabolic substrates that jointly drive the malignant progression of tumors [133, 134]. Glycine is not only a raw material for protein synthesis, but also provides a carbon and nitrogen source for one-carbon unit metabolism and GSH synthesis [135]. After undergoing metabolic reprogramming, cancer cells significantly upregulate glutamine uptake, synthesis, and metabolic utilization to promote their indefinite proliferation [136]. Glutamine is converted to glutamic acid by the enzyme glutaminase (GLS), which is further converted into α-KG. Glutamine is an amino acid that is highly utilized by cancer cells, providing key precursors for anaplerosis of the TCA cycle, nucleotide synthesis, and GSH synthesis [3]. In lung cancer cells, glutamine metabolic reprogramming becomes a key driver supporting cancer cell proliferation and progression [137]. The leucine released by DBT decomposition of BCAA directly activates the mTORC1 pathway, which promotes protein translation and cell proliferation [138].

The metabolic pattern of cancer cells is not static, but dynamically adjusted according to the microenvironment, such as oxygen concentration and nutrient availability, which is called metabolic plasticity. The bidirectional switching of glycolysis and OXPHOS in cancer cells is the core manifestation of metabolic plasticity, which is widely present in a variety of solid tumors and hematological tumors.

## Current Status of Metabolic Reprogramming Therapy for Targeted Cancers

Research targeting metabolic reprogramming of cancer, characterized by the Warburg effect, has made some progress. It is mainly reflected in the inhibition of key enzymes of glycolysis, glucose transporters and lactate production. Lactate dehydrogenase A (LDHA) is a key enzyme in cancer glycolysis that converts pyruvate to lactate and regenerates NAD^+^ [139]. The small molecule inhibitor (FX11 [140]) decreases ATP production by inhibiting LDHA, increases oxidative stress, and induces B lymphocytes death [140]. Glucose transporter 1 (GLUT1) is a key transporter of glucose uptake in cancer cells, and its inhibitors are able to induce cell cycle arrest and inhibit the growth of cancer cells [141]. BAY-876 is a GLUT1 inhibitor that inhibits the growth of a subset of triple-negative breast cancer (TNBC), head and neck squamous cell carcinoma, characterized by high glycolysis and low OXPHOS [142, 143]. BAY-876 has been shown to induce metabolic changes and inhibit cancer cell proliferation in a mouse model of colorectal cancer, but clinical trials have not been conducted due to the uncertain effects of GLUT1 inhibition in humans [144].

Targeting TCA cycles is also challenging in cancer treatment. Coptisine has been shown to specifically inhibit the mitochondrial complex I, block TNBC mitochondrial function, induce apoptosis and inhibit tumor growth in vivo [145]. Other complex I inhibitors (such as IACS-010759) have demonstrated strong inhibitory effects on various cancer cells in vitro, but have encountered severe neurotoxicity in clinical trials, limiting their clinical application [146, 147]. Research has found that drugs based on triphenyl phosphonium cations (TPP^+^) can selectively accumulate in the mitochondria of cancer cells, inhibiting OXPHOS and thus suppressing tumor proliferation. These drugs have shown minimal toxic side effects in rodents and humans [146]. In acute myeloid leukemia (AML), leukemia stem cells (LSCs) are highly dependent on OXPHOS to maintain their survival and function [148]. The LA analogue CPI613 shows strong antitumor activity against AML, NSCLC, pancreatic cancer (PAAD) and by inhibiting the function of PDH [149, 150]. However, the phase III trial of the PDH inhibitor devimistat was terminated prematurely due to ineffectiveness, which is related to AML cells compensating for PDH inhibition through gluconeogenesis [151]. Targeting glutamine metabolism, such as GLS inhibitor CB-839, has antiproliferative activity against TNBC cell lines, but drug resistance remains a therapeutic bottleneck [152, 153].

The abnormal metabolic reprogramming of cancer cells has sparked scientists’ interest in metabolic pathways as potential therapeutic targets. However, existing targeted metabolic therapy strategies still face many challenges. On the one hand, single-agent therapies often have limited efficacy and may cause severe side effects. On the other hand, the metabolic plasticity of cancer cells allows them to adapt to metabolic suppression through compensatory mechanisms, thus weakening the therapeutic effect. These challenges indicate that a single target may be insufficient to achieve the desired therapeutic effect, and multi-target combination therapy strategies may offer new ideas for overcoming these challenges.

Based on the core characteristics of cancer metabolic reprogramming, targeted lipoylation as an anti-cancer strategy has shown unique and promising application potential. Targeting lipoylated proteins can achieve multiple metabolic inhibitions that simultaneously target glycolysis, OXPHOS and amino acid metabolism, which is expected to open up a new strategy in the fight against cancer metabolism.

## Targeted Lipoylation: A New Strategy for Cancer Treatment

Studies have shown that DLAT inhibits leucine decomposition through acetylated AU RNA-binding methylglutaconyl-coenzyme A (CoA) hydratase (AUH) enzymes, leading to the activation of mTOR pathway and promoting hepatocellular carcinoma progression [154]. DLAT is aberrantly expressed in various malignancies such as non-small cell lung cancer (NSCLC) [155], HCC [156], and PAAD [157]. Paredes et al. revealed that under hypoxic conditions in triple-negative breast cancer cells, reduced POLDIP2 expression suppresses lipoylation of critical TCA cycle enzymes DLAT and DLST, consequently impairing oxidative glucose metabolism while enhancing glycolytic flux — a metabolic shift characteristic of the Warburg effect [121]. Importantly, forced POLDIP2 expression restored mitochondrial protein lipoylation, augmenting respiratory capacity and attenuating cancer cell proliferation [121]. These findings establish protein lipoylation as a viable therapeutic target for modulating cancer cell metabolism and growth.

The metabolism of TNBC cells changes from glycolysis to mitochondrial OXPHOS during metastatic spread [158], and similar metabolic alterations occur in chemoresistant TNBC [159]. High DLST expression was observed in TNBC cells and predicted poor overall and recurrence-free survival [69]. Inhibition of KGDH complex activity or depletion of DLST can attenuate TNBC migration and invasion. High-risk neuroblastoma OXPHOS gene upregulation and increased glutamine utilization [160, 161]. High expression of DLST predicts poor survival and disease aggressiveness in neuroblastoma patients [162]. DLST depletion inhibits OXPHOS and reduces NADH levels, and the novel complex I inhibitor IACS-010759 has been shown to inhibit the growth of human neuroblastoma cells in vitro and in vivo1 [162]. The study reveals the potential value of targeting OXPHOS as a treatment for high-risk neuroblastoma. The small molecule inhibitor obatoclax mesylate inhibits the growth of cells by decreasing OXPHOS in cancer cell lines with DLST amplification or high mRNA levels [163]. Similarly, DLST knockdown inhibits the MYC-driven decrease in the viability of T-ALL cells and induces apoptosis [159]. CPI-613 is an LA analogue that blocks TCA circulation and OXPHOS by targeting KGDH and PDH, leading to apoptosis in LSCs due to ATP depletion [150]. In a phase I clinical trial, CPI-613 in combination with chemotherapeutic agents showed promising anti-tumor efficacy in patients with relapsed or refractory AML [150]. In AML, inhibition of OGDH leads to reduced TCA cycle flux, thereby impairing aspartate production and blocking de novo nucleotide biosynthesis, ultimately disrupting AML progression [164]. The small-molecule drug imipridone ONC213 specifically targets KGDH to block the TCA cycle, triggering mitochondrial stress and ultimately activating the apoptotic pathway, providing a novel therapeutic strategy for OXPHOS-dependent AML [165].

In ovarian and breast cancer cell lines, inhibition of BCKDK decreases intracellular BCAA levels and increases cancer cell sensitivity to paclitaxel [166]. In pancreatic ductal adenocarcinoma (PDAC), stromal cells (cancer-associated fibroblasts, CAFs) in the tumor microenvironment exhibit enhanced BCAAs catabolism, and the produced BCKAs are taken up by PDAC cells. cells [167]. As a core subunit of BCKDCH, DBT catalyzes the conversion of BCKAs to branched-chain acyl-CoA, thereby promoting TCA cycle flux and supporting PDAC growth in nutrient-deprived environments. The DBT inhibitor AUH-6 significantly suppresses the progression of high-stroma PDAC models by inhibiting BCKA catabolism and blocking nutrient supply to tumor cells [167]. Russell et al. showed that increased phosphorylation of the BCKDHA subunit in HCC leads to inhibition of BCKDH function and hindrance of BCAA metabolism. The increased BCAAs support the rapid growth of tumor cells by activating the mTORC1 signaling pathway, which promotes cellular anabolism and energy metabolism [86]. Notably, transcriptomic and metabolomic analyses have shown that the expression of the core subunit DBT in BCKDH complex is consistently altered in tumors. Tumor burden in mouse models can be significantly reduced by enhancing BCAA catabolism with the BCKDK inhibitor BT2 or dietary restriction of BCAA accumulation [86]. However, direct evidence of the effect of DBT on tumor growth needs to be confirmed by further specialized studies.

The high expression of GCSH in breast cancer tissues and cells determines the metabolic status and viability of cells, and is a valid tumor marker for breast cancer hyperproliferation [168]. GCSH transcript variants can limit GCSH translation, reduce overall mitochondrial GCS activity, and ultimately lead to plasma membrane damage and necrosis [168]. HCC has been found to have high GCS activity. In addition to providing one-carbon units to support nucleotide synthesis, GCS can also promote tumor growth by maintaining protein lipoylation and mitochondrial activity [116]. GLDC silencing inhibits the lipoylation complex and impairs mitochondrial metabolism, inhibiting tumor growth, which suggests that GCS may be a novel therapeutic target for HCC.

As a key hub of tumor metabolic adaptation, lipoylated enzymes drive malignant progression by regulating energy pathways, amino acid metabolism and redox balance in different cancer types. Targeting the activity level or modification of these enzymes can precisely intervene in the metabolic weakness of cancer cells, providing a new strategy for overcoming drug resistance and developing combination therapies (Table 2). In the future, it is necessary to develop specific agonists/inhibitors based on tumor metabolic heterogeneity and explore precision treatment pathways based on metabolic phenotyping.

**Table 2:** Summary of Lipoylation Pathway Targeting in Cancer Therapies.

Lipoylated protein	Cancers or caner cells	Targeted metabolic type	Mechanism	Targeting strategies	Ref.
DLAT	HCC	leucine decomposition	DLAT Acetylat AUH inhibits leucine decomposition	AUH^K109R^-mRNA lipid nanoparticles	[154]
DLAT	HCC	OXPHOS	Stabilizes mitochondrial function	Elesclomol	[182]
PDH, KGDH	Triple-negative cancer cells	OXPHOS	Increase lipoylated DLAT to improve mitochondrial respiration	Polymerase-δ Interacting protein 2	[121]
DLST	TNBC	OXPHOS	Inhibits TCA cycling	DLST depletion or CPI-613	[69]
DLST	T-ALL	OXPHOS	Disrupts the biochemical conversion of α-KG to succinyl-CoA	DLST knockdown	[70]
DLST	High-risk neuroblastoma	OXPHOS	Inhibits OXPHOS and reduces NADH levels	DLST depletion or complex I inhibitor IACS-010759	[162]
DLST	Cancer cell lines with DLST amplification or high mRNA levels	OXPHOS	Inhibits cellular OXPHOS	Obatoclax mesylate	[163]
DLST, DLAT	AML	OXPHOS	Blocks glucose or glutamine-derived carbon from entering the TCA cycle and promotes PDH phosphorylation	CPI-613	[150]
OGDH	AML	TCA cycle	Impairs the production of aspartic acid	OGDH inhibition	[164]
KGDH	AML	OXPHOS	Targets KGDH to block the TCA cycle	Imipridone ONC213	[165]
BCAA	Ovarian and breast cancer cell lines	BCAA metabolism	Inhibition of BCKDK to decrease intracellular BCAA levels	3,6-dichlorobenzothiophene-2-carboxylic acid (BT2/DCBC) or (S)-2-chloro-4-methylvaleric acid (CMVA)	[166]
BCKDH	HCC	BCAA metabolism	Phosphorylation of the BCKDHA subunit and activation of the mTORC1 signaling pathway	BCKDK inhibitor BT2 or BCAA dietary restriction	[86]
GCSH	Breast cancer cells	Glycine metabolism	Limit GCSH translations	Transcript variants	[168]
GCS	HCC	Glycine metabolism	Inhibits the lipoylation complex	GLDC silence	116]

## The Relationship between Lipoylation and Cuproptosis

Excessive accumulation of copper within cells triggers a new type of programmed cell death called “cuproptosis“ [6]. The core mechanism of cuproptosis is the direct binding of copper to the lipoylated components of the TCA cycle, resulting in lipoylated protein aggregation and loss of iron-sulfur cluster proteins, triggering intense proteinotoxic stress and cell death [6]. Cuproptosis occurs in a manner dependent on intracellular copper ion deposition. Cells take up copper ions via copper transporter 1 (CTR1) and excrete them outside the cell via ATPase transporter (ATP7A/B) [169]. Studies have found that copper and CTR1 have high levels in PAAD, and knockout of CTR1 can inhibit the progression of PAAD [170]. Epigallocatechin gallate (EGCG) promotes intracellular copper accumulation by blocking the transcriptional regulation of ATP7B, thereby enhancing the susceptibility of HCC to cuproptosis [171]. Copper ionophores are compounds that reversibly bind to Cu²⁺ and transfer it into cells to induce cuproptosis. Examples include disulfiram (DSF), NSC-319726, and elesclomol (ES), which have been shown to exert anticancer activity against CRC and glioblastoma [172–174]. The copper chelator triethylenetetramine can reduce intracellular copper deposition, thereby restraining triptolide-induced cuproptosis in cervical cancer cells [175].

**Fig. 4:**
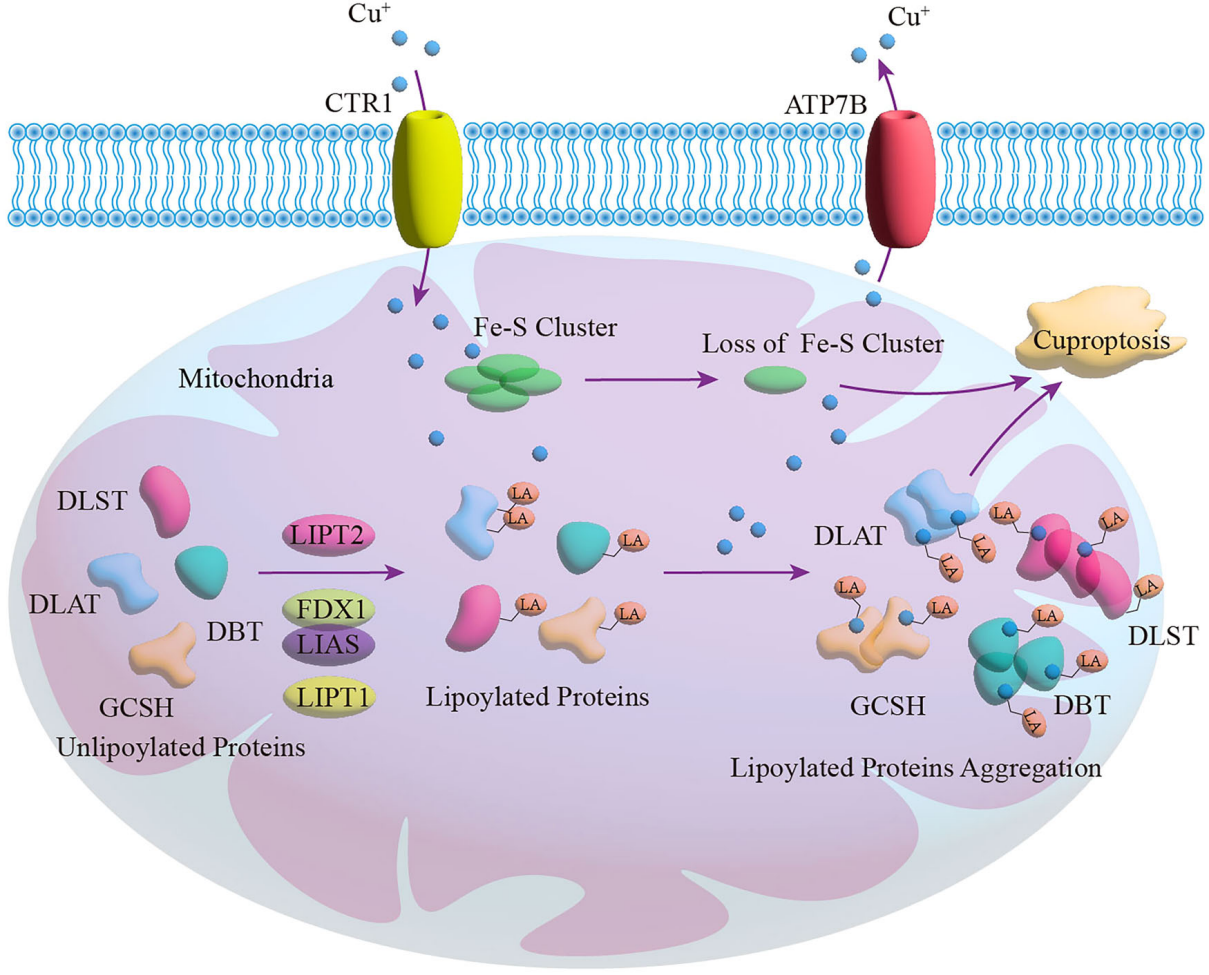
Schematic diagram of cuproptosis and lipoylation. Cu⁺ is transported into the cell via CTR1 and subsequently enters the mitochondria. Key mitochondrial proteins (DLST, DLAT, DBT, GCSH) undergo lipoylation under the catalytic action of enzymes such as LIPT2, FDX1, LIAS, and LIPT1. Cu⁺ directly binds to lipoylated proteins and induces their oligomerization. Additionally, Cu⁺ can also induce the loss of Fe-S clusters. These abnormal processes collectively trigger proteotoxic stress and ultimately lead to cell death.

By dissecting the core mechanisms of cuproptosis and the characteristics of copper accumulation in cancer cells (Fig. 4), previous study revealed that lipoylated proteins serve as the central hub connecting cuproptosis to the vulnerability of cancer cells [6]. Targeting lipoylated proteins to disrupt their key metabolic functions can specifically attack these metabolically abnormal cancer cells. Therefore, interfering with the lipoylation process or specifically targeting lipoylated proteins to enhance cancer cells’ sensitivity to copper ions provides a promising approach for efficiently and selectively killing cancer cells. In addition, copper ionophores can efficiently deliver exogenous copper to the mitochondria of cancer cells, significantly enhancing the toxic effect on lipoylated proteins [176]. The combination of targeting lipoylated proteins and copper ionophores is expected to induce cuproptosis synergistically.

FDX1 acts as a core regulator of cuproptosis and serves as the direct target of the copper ionophore elesclomol-Cu complex (ES-Cu) [6]. FDX1 reduces Cu²⁺ to the more toxic Cu¹⁺, facilitating the binding of copper ions to lipoylated proteins and thereby increasing cellular sensitivity to cuproptosis. Furthermore, FDX1 acts as an upstream regulator of lipoylation, with its knockout suppressing cuproptosis by disrupting the lipoylation of DLST and DLAT [6]. Therefore, targeting FDX1 is expected to enhance the sensitivity of cancer cells to cuproptosis. New research has found that knockdown of FDX1 reduces the level of lipoylation of DLAT and DLST in thyroid cancer cells, and copper-induced cancer cell death is inhibited [177]. Pan-cancer analysis shows that knocking down FDX1 leads to the downregulation of cuproptosis in clear cell renal tumor cells [178]. Emerging research has shown that knockdown of FDX1 decreases the lipoylation levels of DLAT and DLST in thyroid cancer cells, inhibiting copper-induced cancer cell death [177]. High copper content in gastric cancer tissues promotes lactylation of methyltransferase-like protein 16 (METTL16) and ultimately induces cuproptosis in GC cells by upregulating FDX1 protein levels [179]. The combination of the SIRT2 inhibitor AGK2 and elesclomol promotes cuproptosis in gastric cancer cells by enhancing the lactylation of METTL16 at lysine 229 and increasing FDX1 protein levels [179]. In TNBC, excessive copper activates AKT1, which inhibits protein lipoylation by phosphorylating FDX1 [180]. This ultimately suppresses cuproptosis, leading to resistance of TNBC cells to cuproptosis. The combination of the AKT1 inhibitor MK2206 and elesclomol has been demonstrated to synergistically promote cuproptosis and inhibit tumorigenesis in TNBC both in vitro and in vivo [180].

DLAT serves as the direct target of cuproptosis. By regulating its expression and functional state, targeted intervention in cuproptosis can be achieved. Patients with high expression of DLAT in HCC cells and PAAD cells respond better to immunotherapy [157]. In clear cell renal cell carcinoma (ccRCC), which is predominantly glycolyzed, DLAT expression levels are lower than in adjacent tissues [181]. Patients of ccRCC with low DLAT expression have lower survival rates, poor clinical prognosis, and more substantial immune cell infiltration [181]. In HCC, the highly expressed maternal embryonic leucine zipper kinase (MELK) upregulates DLAT through activating the PI3K/mTOR pathway, stabilizing mitochondrial function and accelerating the progression of HCC [182]. Conversely, elesclomol treatment significantly suppresses HCC proliferation by increasing DLAT oligomerization to induce cuproptosis [182]. Studies have shown that DLAT is highly expressed in TNBC. DLAT directly binds to Yes-Associated Protein 1 (YAP1), inhibits phosphorylation, and promotes nuclear translocation, thereby regulating the expression of downstream oncogene connective tissue growth factor and ultimately accelerating TNBC progression [183]. The YAP1 inhibitor verteporfin reverses the proliferation of TNBC induced by DLAT overexpression by binding to YAP1 and inhibiting its transcriptional activity [183]. In ccRCC and PTC cells, hypoxia activates PDK via HIF-1α. PDK then phosphorylates DLAT and promotes its ubiquitination [184]. The reduction of DLAT leads to resistance to cuproptosis in cancer cells under hypoxic conditions. The HIF-1α inhibitor PX-478 blocks the activation of PDK, thereby increasing DLAT expression levels [184]. The combination of PX-478 and ES-Cu can amplify copper ions’ toxicity and promote cuproptosis [184].

These studies reveal a close association between cuproptosis and the lipoylation pathway. Targeted regulation of lipoylation modification can serve as a key strategy to enhance anti-tumor efficacy by increasing cancer cells’ sensitivity to cuproptosis. Specifically, upregulation of lipoylated proteins increases cancer cells’ sensitivity to copper ion-induced cell death. Given that lipoylated proteins are directly involved in mitochondrial metabolism, inducing cuproptosis can effectively suppress tumor cells dependent on high-level aerobic respiration, such as cancer stem cells, melanoma, breast cancer, and drug-resistant tumors [174, 185–188].

Ferroptosis is a programmed form of cell death caused by iron-catalyzed lipid peroxidation, characterized by elevated intracellular iron levels and accumulation of lipid peroxides [189]. Comparing ferroptosis and cuproptosis, it is interesting to note that glutathione (GSH) acts at the crossing point of the regulation network, therefore, GSH may be a key aspect of the therapeutic target. Recently, we found that DSF/Cu induced both ferroptosis and cuproptosis, accompanied by the depletion of GSH, elevation of lipid peroxides, and compensatory elevation of xCT [190]. Inhibiting the compensatory increase of xCT renders HCC cells more susceptible to DSF/Cu. This may provide a promising synergistic strategy to sensitize tumor therapy and overcome drug resistance, as it activates different programmed cell death [190]. Additionally, we demonstrated that GSH exhaustion via inhibition of the xCT-GSH-GPX4 pathway synergistically enhanced DSF/Cu-induced cuproptosis in myelodysplastic syndromes [191]. The dissociative components of the cascade nanoreactor CaCuZC constructed by Tang et al. simultaneously induce both cuproptosis and ferroptosis, and cause intracellular GSH depletion and lipid peroxidation accumulation [192]. This synergistic therapy activates powerful immunogenic cell death, thereby improving the efficacy of tumor immunotherapy. Although cuproptosis and ferroptosis exhibit distinct initiation mechanisms and molecular signatures, emerging evidence reveals significant crosstalk between these two regulated cell death pathways. This interplay adds complexity to their regulatory networks and presents novel opportunities for combinatorial cancer therapy. Strategic integration of both death modalities may synergistically enhance therapeutic efficacy while overcoming monotherapy resistance. While this field remains in its early stages, further mechanistic studies and preclinical validation are warranted to fully exploit its translational potential.

## Conclusions and Future Prospects

In humans, four lipoylated proteins have been identified as core components of key metabolic enzymes involved in glycolysis, the TCA cycle, and amino acid metabolism. The dynamic addition or removal of lipoylation modifications critically regulates the functional activity of these enzymes, underscoring their essential role in maintaining metabolic homeostasis. Although cancer metabolic reprogramming has been well studied, there is little literature on the effects of lipoylated protein on cancer cell metabolism. Lipoylated proteins precisely coordinate the activities of key metabolic nodes, including PDH, KGDH, BCKDH and GCS. By acting as central hubs of metabolic regulation, they dynamically rewire the energy and biosynthetic networks of cancer cells. These enzyme complexes directly control TCA cycle flux, redox homeostasis, and precursor supply for biosynthetic pathways through lipoylated modifications. Functional perturbations of these complexes trigger mitochondrial metabolic reprogramming, offering novel therapeutic vulnerabilities for targeting cancer metabolism. In-depth elucidation of their regulatory mechanisms advances fundamental understanding of cancer metabolic reprogramming and establishes a molecular foundation for developing innovative anti-cancer strategies targeting metabolic nodes.

Recent studies have elucidated the mechanism of cuproptosis through the targeting of lipoylated proteins, offering valuable insights into the role of these proteins in cell death. Cuprotosis relies on mitochondrial respiration, and lipoylated proteins serve as direct targets of copper toxicity. By intervening in the lipoylation modification process, the metabolic homeostasis of cancer cells can be precisely disrupted, thereby inducing their high sensitivity to copper ion toxicity. On this basis, the application of copper ionophores has demonstrated significant advantages. Such carriers can efficiently mediate the directional transport of exogenous copper ions to the mitochondria of cancer cells, thus greatly enhancing the toxic effect of copper ions on lipoylated proteins. The combination of lipoylated protein-targeted therapy and copper ionophores can form a powerful synergistic effect, effectively triggering cuprotosis through multiple action mechanisms. This has opened up a brand-new technical path for the precise treatment of tumors. Recent studies demonstrate that copper nanoparticles potentiate antitumor effects through dual induction of cuproptosis and ferroptosis by disrupting glutathione biosynthesis, revealing a novel synergistic mechanism in cancer therapy. Our experiments further demonstrated that inhibiting the xCT-GSH-GPX4 pathway can synergistically enhance disulfiram/copper (DSF/Cu)-induced cuproptosis in myelodysplastic syndrome [191]. These findings propose a promising synergistic strategy for developing novel combination therapies through the concurrent activation of ferroptosis and cuproptosis.

While current research highlights lipoylation as a promising therapeutic target for cancer, the precise mechanisms by which lipoylated proteins operate in cancer cells remain incompletely elucidated. Recent breakthroughs in cancer biology, particularly the discovery of cuproptosis, have deepened our understanding of tumorigenesis and uncovered novel molecular pathways linking lipoylation to programmed cell death. These insights not only reveal the intricate interplay between lipoylation and cancer progression but also open new avenues for developing targeted therapies.

Emerging evidence suggests that dysregulated lipoylation contributes to diverse pathophysiological conditions, with its modulation holding therapeutic potential beyond oncology. Future investigations in this rapidly evolving field must prioritize addressing critical knowledge gaps, including the spatiotemporal regulation of lipoylation in tumor microenvironments and its crosstalk with other PTM systems. Such advances will establish a robust scientific framework for translating lipoylation-related discoveries into clinical strategies, ultimately enabling precision interventions for treating and preventing diseases associated with metabolic reprogramming and mitochondrial dysfunction.
